# Suppression of Notch Signaling Stimulates Progesterone Synthesis by Enhancing the Expression of NR5A2 and NR2F2 in Porcine Granulosa Cells

**DOI:** 10.3390/genes11020120

**Published:** 2020-01-22

**Authors:** Rihong Guo, Fang Chen, Zhendan Shi

**Affiliations:** 1 Jiangsu Key Laboratory for Food Quality and Safety-State Key Laboratory Cultivation Base of Ministry of Science and Technology, Jiangsu Academy of Agricultural Sciences, Nanjing 210014, China; rhguo@jaas.ac.cn; 2 Institute of Animal Science, Jiangsu Academy of Agricultural Sciences, Nanjing 210014, China; fchen_m@sina.com

**Keywords:** porcine, granulosa cells, progesterone, Notch signaling, NR5A2, NR2F2

## Abstract

The conserved Notch pathway is reported to be involved in progesterone synthesis and secretion; however, the exact effects remain controversial. To determine the role and potential mechanisms of the Notch signaling pathway in progesterone biosynthesis in porcine granulosa cells (pGCs), we first used a pharmacological γ-secretase inhibitor, *N*-(*N*-(3,5-difluorophenacetyl-l-alanyl))-S-phenylglycine t-butyl ester (DAPT), to block the Notch pathway in cultured pGCs and then evaluated the expression of genes in the progesterone biosynthesis pathway and key transcription factors (TFs) regulating steroidogenesis. We found that DAPT dose- and time-dependently increased progesterone secretion. The expression of steroidogenic proteins NPC1 and StAR and two TFs, NR5A2 and NR2F2, was significantly upregulated, while the expression of HSD3B was significantly downregulated. Furthermore, knockdown of both *NR5A2* and *NR2F2* with specific siRNAs blocked the upregulatory effects of DAPT on progesterone secretion and reversed the effects of DAPT on the expression of NPC1, StAR, and HSD3B. Moreover, knockdown of NR5A2 and NR2F2 stimulated the expression of Notch3. In conclusion, the inhibition of Notch signaling stimulated progesterone secretion by enhancing the expression of NPC1 and StAR, and the two TFs NR5A2 and NR2F2 acted as downstream TFs of Notch signaling in regulating progesterone synthesis.

## 1. Introduction

Progesterone plays crucial roles in regulating the estrous cycle, the maintenance of pregnancy, and the success of female reproduction [1]. It is mainly secreted by the large lutein cells of the corpora lutea (CL) in the ovary, which are derived from granulosa cells (GCs) after ovulation [1]. Steroidogenic proteins such as steroidogenic acute regulatory protein (StAR), low-density lipoprotein receptor (LDLR), scavenger receptor class b member 1 (SRB1), NPC intracellular cholesterol transporter 1 (NPC1), and sterol carrier protein 2 (SCP2), which are involved in the uptake and transport of cholesterol, and two enzymes, namely, cholesterol side-chain cleavage enzyme (Cyp11a1) and 3-β-hydroxy-delta (5)-steroid dehydrogenase (HSD3B), which process cholesterol to progesterone, are highly expressed in GCs and the CL [2,3]. Progesterone secretion and the expression of the steroidogenic protein in the CL and GCs are tightly regulated by hormones and cytokines through several signaling pathways, such as the LH/PKA, PI3K/AKT, TGFβ, and Notch signaling pathways [1,4,5].

The Notch signaling pathway is an evolutionarily conserved and contact-dependent signaling system [4]. The canonical Notch pathway is composed of four Notch receptors (Notch 1–4) and five Notch ligands (delta-like (Dll)-1, Dll3, Dll4, Jagged1, and Jagged2). Both the Notch receptors and ligands are membrane proteins [6]. Binding of a ligand to its receptor makes the Notch receptor susceptible to ADAM metalloproteases and γ-secretase catalyzed proteolytic cleavages, which releases the intracellular domain of Notch (NICD) into the cytoplasm [6]. After translocation to the nucleus, NICD associates with other TFs, thus regulating the transcription of Notch effector target genes, such as hairy/enhancer of split (Hes) and the hairy-related transcription factor (Hey) genes [6].

In ovary, Notch components and effectors are differentially and dynamically expressed during follicle and CL development [4]. Notch signaling plays important roles in ovarian functions, such as follicle assembly and growth, oocyte meiotic maturation, ovarian vasculogenesis and steroidogenesis [4]. Conditional knock out of *Notch2* and *Notch3* genes in GCs results in multioocyte follicles due to a lack of granulosa cell proliferation [7,8], indicating its essential role in primordial follicle formation [9]. In contrast to its acknowledged role in follicular development, the effects of Notch signaling on steroidogenesis remains controversial. Both suppressive [10,11] and stimulatory [12,13,14] effects of Notch signaling on steroidogenesis have been reported. George, et al. [11] and Wang, et al. [10] reported that Notch signaling plays a suppressive role in progesterone biosynthesis and the expression of steroidogenic protein in GCs. Prasasya and Mayo [12] reported that knockdown of Notch ligand *Jagged1* in murine GCs resulted in reduced progesterone biosynthesis, indicating a stimulatory effect of Notch signaling on steroidogenesis.

The study of Notch signaling in porcine is rare. Notch signaling was reported to stimulate the proliferation of porcine satellite cells [15,16], inhibit adipogenesis of porcine mesenchymal stem cells [17], and protect oxidative stress-induced apoptosis of aortic endothelial cells [18]. However, the effects of Notch signaling on the function of the porcine ovary, such as steroidogenesis, have not been studied.

In brief, progesterone is an important hormone for female reproduction, and its secretion is regulated by the Notch signaling pathway in human and model organisms. However, the effects of Notch singling on steroidogenesis remain controversial in the literature. Moreover, the effects of Notch signaling on steroidogenesis in the porcine ovary have not yet been reported. Thus, this study was designed to resolve the potential role and the underlying mechanisms of Notch signaling in the regulation of progesterone biosynthesis in pGCs.

## 2. Materials and Methods

### 2.1. Cell Culture

Primary porcine granulosa cells (pGCs) were cultured as in our previous studies [19,20]. Briefly, pGCs were isolated from ovaries obtained from a local slaughterhouse. pGCs were plated in culture plates at 1 × 10^6^ cells/mL with cell culture medium (DMEM/F12 with 10% FBS, 100 IU/mL of penicillin, and 100 µg/mL of streptomycin) and incubated in a humidified atmosphere with 5% CO_2_ at 37 °C. Non-adherent cells were gently removed after 24 h by changing the medium. The adherent cells were treated with DAPT (S2215, Selleck, Shanghai, China).

### 2.2. siRNAs and Cell Transfection

Cells in 12- or 6-well plates were transfected with siRNA for NR5A2 (siNR5A2, sense: 5′- CGGAGGAAUACCUGUACUATT-3′), NR2F2 (siNR2F2, sense: 5′-CCGUAUAUGGCAAUUCAAUTT-3′), or scramble siRNA (siNC) using Lipofectamine 3000 (L3000015, Thermo Fisher Scientific, Shanghai, China) according to the manufacturer’s protocol. Cells in 12- and 6-well plates were transfected with 10 nM siRNA or 20 nM siRNA, respectively. Six hours after transfection, the medium was changed to medium with or without DAPT, and the pGCs were cultured for a further 48 h.

### 2.3. RT-qPCR Assay

Total RNA was extracted using RNA isolator (R401-01, Vazyme, Nanjing, China) and reverse-transcribed into cDNA using a HiScript qRT SuperMix with gDNA Eraser (R122-01, Vazyme) according to the manufacturer’s instructions. RT-qPCR was performed on an ABI 7500 (Applied Biosystems, Shanghai, China) using the ChamQ SYBR qPCR Master Mix (Q311-02, Vazyme, Nanjing, China). Primers for RT-qPCR are listed in Supplemental Table S1. Gene expression levels were calculated using the 2^−ΔΔCt^ method and normalized to β-actin mRNA expression.

### 2.4. Western Blot Analysis

Total protein was extracted using RIPA lysis buffer (P0013B, Beyotime Biotechnology, Nantong, China) with phosphatase inhibitor cocktail C (P1091, Beyotime Biotechnology). Then, the cell lysates were boiled in the gel-loading buffer, and 30 μg of protein was separated by SDS-PAGE in each lane of a 12% gel. The proteins were subsequently transferred to a polyvinylidene fluoride membrane (Millipore, Darmstadt, Germany) and probed with primary antibodies against StAR (DF6192, Affinity Biosciences, Changzhou, China), Cyp11a1 (DF6569, Affinity Biosciences), HSD3B (DF6639, Affinity Biosciences), ERK1/2 (4695T, Cell Signaling Technology, Danvers, MA, USA), pERK1/2 (4370T, Cell Signaling), NR2F2 (NBP1-31980, Novus Biologicals, CO, USA), NR5A2 (NBP2-27196SS, Novus) and β-actin (20536-1-AP, Protein Tech, Wuhan, China). Chemiluminescence was detected by an ECL kit from Pierce Chemical (Dallas, Texas, USA) and visualized through Image Quant LAS 4000 (Fujifilm, Tokyo, Japan). Band intensity was quantified with ImageJ software (NIH, Bethesda, MA, USA).

### 2.5. ELISA

Progesterone in the culture medium was measured using an enzyme-linked immunosorbent assay (ELISA) kit (Beijing North Institute of Biological Technology) according to the manufacturer’s instructions. The standard curve of the kit ranged from 0 to 30 ng/mL. Inter- and intra-assay coefficients of variation for these assays were less than 10%. Each sample was measured in triplicate.

### 2.6. Statistical Analysis

Data are presented as the mean ± SEM, where *p* < 0.05 was considered to be significant. All the experiments were performed using a minimum of three biological replicates. Differences between groups were calculated with GraphPad Prism 8 (GraphPad Software, Inc., San Diego, CA, USA).

## 3. Results

### 3.1. DAPT Treatment Stimulated Progesterone Secretion in pGCs

**Figure 1:**
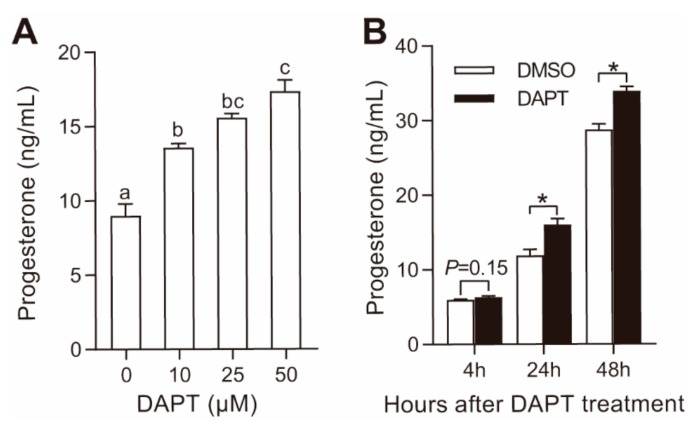
Effects of *N*-(*N*-(3,5-difluorophenacetyl-l-alanyl))-S-phenylglycine t-butyl ester (DAPT) treatment on progesterone secretion in porcine granulosa cells (pGCs). Dose-dependent effect (**A**) and time-course effect (**B**) of DAPT on progesterone secretion in pGCs. Primary pGCs were treated with a Notch pathway inhibitor, DAPT, at varying concentrations (0, 10, 25, and 50 μM DAPT for 48 h) and for various times (4, 24, and 48 h with 25 μM DAPT). Different letters in (**A**) and * in (**B**) mean significant differences at *p* < 0.05.

As reported in model organisms, Notch pathway proteins are expressed in pGCs (Figure S1A). In our previous whole-genome transcriptional study, the most abundant Notch receptor, ligands, and effectors in pGCs were Notch2, Notch3, Jagged1, Hes1, Hes6, and Hey2, respectively (Table S2). To better understand the functions of the Notch pathway in steroidogenesis, DAPT was used to block Notch pathway in pGCs in this study. As shown in Figure 1A, DAPT dose-dependently stimulated progesterone secretion in pGCs after incubation for 48 h. Regarding the time-course effect, progesterone in the culture medium tended to increase 4 h after treatment with 25 μM DAPT and significantly increased after 24 h and 48 h (Figure 1B). After DAPT (25 μM) treatment, the expression of Notch effectors Hes1 and Hey2 was significantly downregulated (Figure S1B). Similar to the results in the mouse ovary and granulosa cells [12], DAPT treatment significantly induced the phosphorylation of ERK1/2 (Figure S1C). These results indicated that DAPT treatment inhibited Notch signaling and stimulated progesterone secretion in pGCs.

### 3.2. DAPT Treatment Altered the Expression of Genes Involved in Steroidogenesis

**Figure 2:**
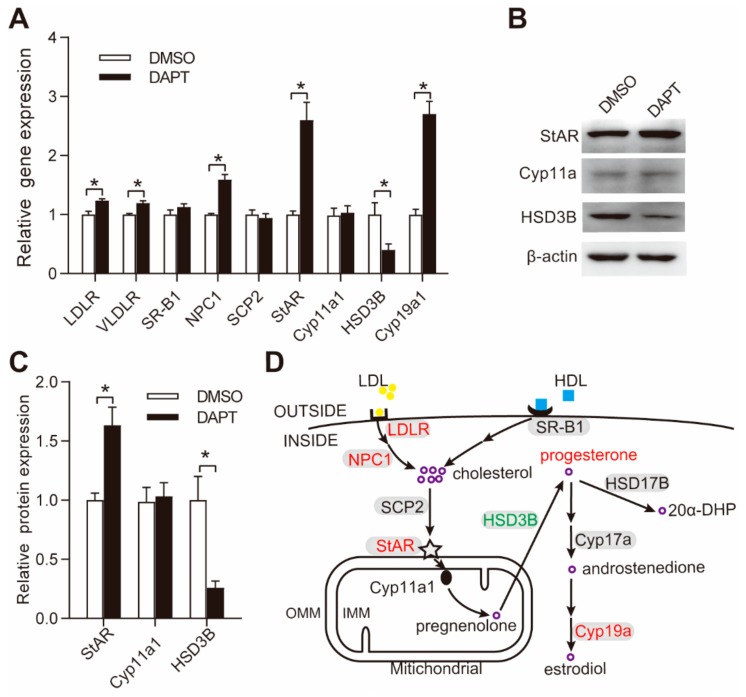
Effects of DAPT treatment on the expression of proteins involved in steroidogenesis in pGCs. (**A**) The gene expression of factors involved in cholesterol uptake (LDLR, VLDLR, SRB1, NPC1, SCP2) and progesterone synthesis (StAR, Cyp11a1, HSD3B); (**B**,**C**) The protein expression of StAR, Cyp11a1, and HSD3B; (**D**) Graphical summary of the gene expression of factors in the progesterone biosynthesis pathway. The upregulated proteins (LDLR, NPC1, StAR, Cyp19a1) are highlighted in red, while the downregulated protein (HSD3B) is highlighted in green; the proteins that remained unchanged or undetected are in black. OMM, outer mitochondrial membrane; IMM, inner mitochondrial membrane; LDL, low-density lipoprotein; HDL, high-density lipoprotein; LDLR, low-density lipoprotein receptor; SRB1, scavenger receptor class b member 1; NPC1, NPC intracellular cholesterol transporter 1; SCP2, sterol carrier protein 2; StAR, steroidogenic acute regulatory protein; HSD17B1, hydroxysteroid 17-β dehydrogenase 1; Cyp17a, steroid 17-α-hydroxylase; Aromatase, Cyp19a1. Primary pGCs were treated with 25 μM DAPT for 48 h. * means significant difference at *p* < 0.05.

We next evaluated the expression of steroidogenic proteins, such as LDLR, VLDLR, SRB1, NPC1, SCP2, StAR, Cyp11a1, HSD3B, and aromatase (Cyp19a1). The gene expression of *LDLR*, *VLDLR*, *NPC1 StAR*, and *Cyp19a1* was significantly upregulated, while the gene expression of *HSD3B* was significantly downregulated (Figure 2A). The expression of SRB1, SCP2, and Cyp11a1 remained unchanged (Figure 2A). The protein expression of StAR, Cyp11a1, and HSD3B was significantly upregulated, unchanged and downregulated, respectively, which was similar to their gene expression (Figure 2B,C). The accumulation of progesterone depends not only on biosynthesis but also metabolism. However, the expression of HSD17B and Cyp17a1, the two main enzymes involved in progesterone metabolism (Figure 2D), was relatively low in GCs without LH/PKA stimulation, and the metabolism of progesterone could be ignored in pGCs. Thus, the effects of DAPT treatment on the expression of genes involved in steroidogenesis can be summarized as shown in Figure 2D.

### 3.3. DAPT Treatment Stimulated the Expression of NR5A2 and NR2F2

**Figure 3:**
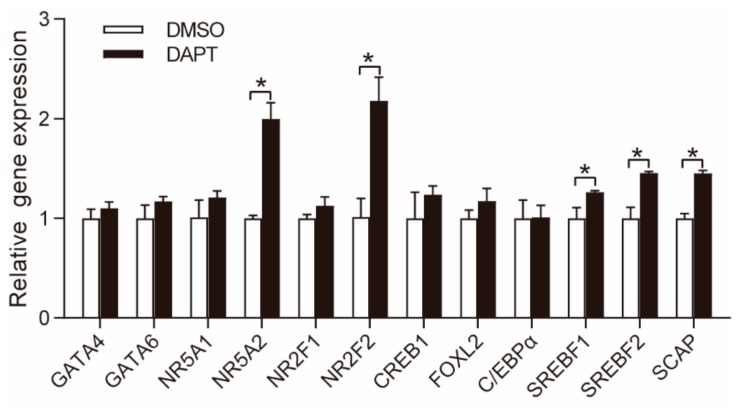
Effects of DAPT treatment on the gene expression of key TFs involved in steroidogenesis. Primary pGCs were treated with 25 μM DAPT for 48 h. * means significant difference at *p* < 0.05.

GATA binding protein 4 (GATA4), GATA binding protein 6 (GATA6), nuclear receptor subfamily 5 group a member 1 (NR5A1), nuclear receptor subfamily 5 group a member 2 (NR5A2), nuclear receptor subfamily 2 group F member 1 (NR2F1), nuclear receptor subfamily 2 group F member 1 (NR2F2), cAMP responsive element binding protein 1 (CREB1), forkhead box L2 (FOXL2), CCAAT/enhancer binding protein α (C/EBPα), sterol regulatory element binding transcription factor 1 (SREBF1), sterol regulatory element binding transcription factor 2 (SREBF2), and SREBP cleavage-activating protein (SCAP) are important TFs involved in steroidogenesis by regulating the expression of steroidogenic proteins [21,22,23,24,25,26]. In this study, the mRNA expression of *NR5A2* (1.8-fold), *NR2F2* (2.4-fold), *SREBF1* (1.2-fold), *SREBF2* (1.4-fold), and SCAP (1.4-fold) was significantly upregulated, while the expression of *GATA4*, *GATA6, NR5A1*, *CREB1*, and *C/EBPα* remained unchanged (Figure 3).

NR5A2 and NR2F2 are reported to reciprocally repress Notch signaling in neuronal cells [27] and mouse testis [28], respectively. In this study, the expression of these two TFs was significantly upregulated after blockade of Notch pathway in pGCs. We speculated that NR5A2 and NR2F2 were potential mediators of Notch signaling in regulating steroidogenesis, and the interactions between the two TFs and Notch signaling in progesterone synthesis were further studied using specific siRNAs to knockdown these two TFs. SREBF1, SREBF2, and SCAP were not considered to be potential mediators of Notch pathway due to the moderate increase in gene expression.

### 3.4. Effects of NR5A2 Knockdown on Progesterone Secretion in pGCs

**Figure 4:**
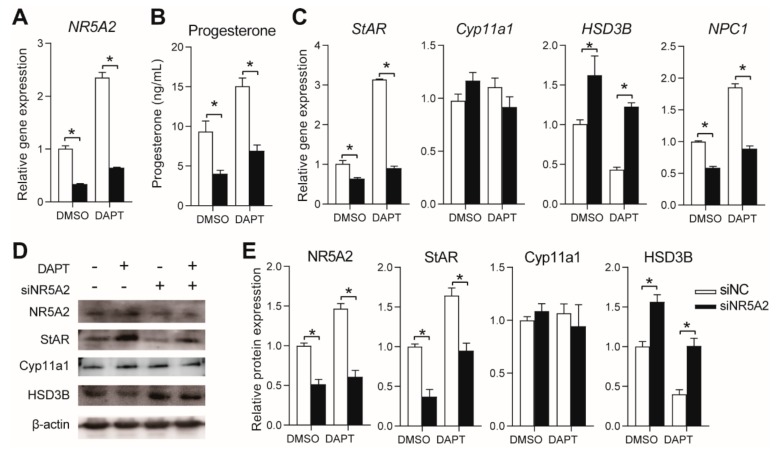
Effects of NR5A2 knockdown on progesterone biosynthesis. (**A**) gene expression of NR5A2 after siRNA transfection; (**B**) effects of NR5A2 knockdown on progesterone secretion; (**C**) gene expression of key factors (StAR, Cyp11a1, HSD3B, and NPC1) involved in progesterone biosynthesis; (**D**,**E**) protein expression of NR5A2, StAR, Cyp11a1, and HSD3B. Primary pGCs were transfected with NR5A2 siRNA (siNR5A2) or scramble siRNA (siNC). Six hours later, the medium was changed to fresh medium with DMSO or DAPT, followed by further culture for 48 h. * means significant difference at *p* < 0.05.

After specific siRNA (siNR5A2) transfection, the gene and protein expression of *NR5A2* was significantly reduced compared with the scramble siRNA treatment (siNC) in both the DMSO- and DAPT-treated cells (Figure 4A,D,E). Progesterone secretion was significantly downregulated after *NR5A2* knockdown, and the stimulatory effect of DAPT on progesterone secretion was reversed by *NR5A2* knockdown (Figure 4B). NR5A2 knockdown significantly downregulated the expression of NPC1 and StAR and upregulated the expression of HSD3B, while the expression of Cyp11a1 remained unaltered. With DAPT treatment, *NR5A2* knockdown blocked *StAR* and *NPC1* upregulation and reversed HSD3B downregulation (Figure 4C). The protein expression levels of *StAR*, *Cyp11a1,* and *HSD3B* were similar to their mRNA expression patterns (Figure 4D,E).

### 3.5. Effects of NR2F2 Knockdown on Progesterone Secretion in pGCs

**Figure 5:**
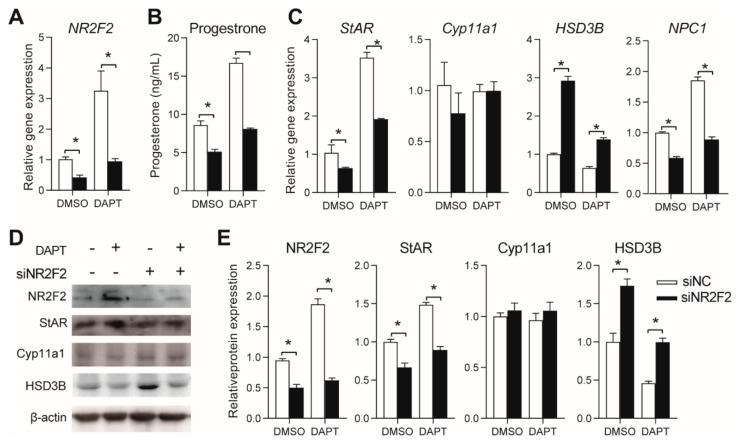
Effects of NR2F2 knockdown on progesterone biosynthesis. (**A**) gene expression of NR2F2 after siRNA transfection; (**B**) Effects of NR2F2 knockdown on progesterone secretion; (**C**) gene expression of key factors (StAR, Cyp11a1, HSD3B, and NPC1) involved in progesterone biosynthesis; (**D**,**E**) protein expression of NR5A2, StAR, Cyp11a1, and HSD3B. Primary pGCs were transfected with NR2F2 siRNA (siNR2F2) or scramble siRNA (siNC). Six hours later, the medium was changed to fresh medium with DMSO or DAPT, followed by further culture for 48 h. * means significant difference at *p* < 0.05.

The gene and protein expression of *NR2F2* was significantly reduced after specific siRNA (sNR2F2) treatment compared with that after siNC treatment in both DMSO- and DAPT-treated cells (Figure 5A,D,E). After *NR2F2* knockdown, progesterone secretion was significantly downregulated in both the DMSO- and DAPT-treated cells, and the stimulatory effect of DAPT on progesterone secretion was reduced to the level of that in untreated cells (Figure 4B). NR2F2 knockdown significantly downregulated the gene expression of *StAR* and *NPC1* and upregulated the expression of HSD3B (Figure 4C–E). The effects of DAPT on the gene expression of *StAR*, *NPC1*, and *HSD3B* were reversed by NR2F2 knockdown (Figure 4C–E). Neither DAPT nor NR2F2 knockdown affected *Cyp11a1* expression (Figure 4C–E).

The expression of SRB1 rather than LDLR and VLDLR was downregulated after NR5A2 and NR2F2 knockdown (Figure S2), indicating positive roles of NR5A2 and NR2F2 on the expression of SRB1. These results also indicated that upregulation of LDLR and VLDLR expression after DAPT treatment may not be caused by the upregulated expression of NR5A2 and NR2F2; other mechanisms for the regulation of LDLR and VLDLR expression via Notch signaling may exist.

### 3.6. Effects of NR5A2 and NR2F2 Knockdown on the Gene Expression of Notch Proteins

To investigate whether NR5A2 and NR2F2 inhibited Notch signaling in pGCs, we assessed the expression of Notch receptors and ligands after NR5A2 and NR2F2 interference. Among the detected Notch receptors and ligands, only the expression of Notch3 was upregulated after NR5A2 and NR2F2 knockdown, while the expression of Notch2, Dll1, and Jagged1 remained unchanged. These results indicated that Notch3 rather than Notch2 was inhibited by NR5A2 and NR2F2, and Notch 3 may be a target gene of NR5A2 and NR2F2 in pGCs.

**Figure 6:**
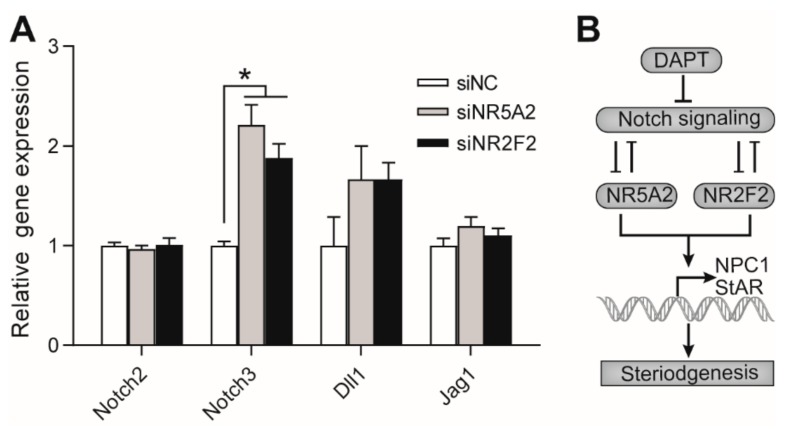
Effects of NR5A2 and NR2F2 knockdown on the expression of Notch receptors and ligands. (**A**) Effects of NR5A2 and NR2F2 on the expression of Notch receptors (Notch2 and Notch3) and Notch ligands (Dll1 and Jagged1). Primary pGCs were transfected with NR5A2 siRNA (siNR5A2), NR2F2 siRNA (siNR2F2), or scramble siRNA (siNC). (**B**) Schematic representation of the Notch/NR5A2/NR2F2 pathway in regulating steroidogenesis pGCs. * means significant difference at *p* < 0.05.

The results of this study can be summarized as shown in Figure 6B. In brief, Notch signaling mutually inhibited NR5A2 and NR2F2, and the blockade of Notch singling by DAPT treatment significantly stimulated the expression of NR5A2 and NR2F2. Both NR5A2 and NR2F2 stimulated the expression of NPC1 and StAR, which in turn enhanced progesterone biosynthesis after DAPT treatment in pGCs.

## 4. Discussion

In this study, we found that the Notch pathway inhibited progesterone secretion in pGCs and that disruption of Notch signaling by DAPT treatment significantly stimulated the expression of NPC1, StAR, NR2F2, and NR5A2. To the best of our knowledge, this is the first study focusing on the effect of Notch pathway on steroidogenesis in pGCs, and we identified NR2F2 and NR5A2 as potential downstream TFs of Notch signaling in regulating progesterone biosynthesis.

Notch signaling regulates cell fate by controlling cell proliferation and differentiation [29], and it is required for GC proliferation and follicle development in ovary [7,9,30]. Conditional knockout of *Notch2* and *Jagged1* in ovary results in the failure of GC proliferation [7]. In an ex vivo ovary and follicle culture system, inhibition of Notch signaling resulted in loss of granulosa cell proliferation [9,30]. It seems that the Notch pathway promotes the proliferation of GCs and may repress the terminal differentiation of GCs into luteal cells, which are the major source of progesterone in the estrous cycle. In other words, Notch signaling may impede luteinization of GCs. To this extent, it is easy to interpret the inhibitory effects of Notch signaling on progesterone secretion observed in our study.

The inhibitory effects of the Notch pathway on progesterone secretion from GCs have also been previously reported in model organisms. George, et al. [11] found that inhibition of Notch signaling by DAPT stimulated progesterone secretion in murine GCs and Leydig cells. Wang, et al. [10] reported that activation of Notch signaling by the overexpression of NICD2 reduced progesterone biosynthesis in murine GCs. In contrast, positive effects of the Notch pathway on progesterone secretion have also been reported. In murine GCs, knockdown of *Jagged1* reduced progesterone biosynthesis [12], and the in vivo and in vitro administration of DAPT to CL tissue to suppress Notch signaling decreased the progesterone concentration in the serum of pregnant rats and culture medium, respectively [13,14]. The observed controversial results may be due to two reasons. First, previous studies have shown that different Notch ligands could have different, or even opposite, effects [31,32]. For example, in growing blood vessels, angiogenesis is inhibited and stimulated by the Notch ligands Dll4 and Jagged1, respectively [31]. The mechanisms related to the different Notch ligands resulting in opposite effects may also apply to other Notch-controlled biological processes, such as steroidogenesis in GCs and CLs. Second, Notch ligands and receptors are dynamically expressed during follicle and CL development [33]. Taking a study in mice as an example, Jagged1 is expressed in the GCs of primary and secondary follicles and CLs but not in the GCs of the antral follicle. Thus, the apparent inconsistencies among studies may be related to the use of different study materials (GCs or CL tissue) or the use of different means of modulating Notch signaling (Notch receptor knockdown, NICD overexpression, or Notch ligand knockdown).

The upregulation of progesterone secretion after DAPT treatment was mainly caused by the increased expression of steroidogenic proteins NPC1 and StAR in this study. NPC1 is a late endosomal protein and is required for the delivery of LDL-derived cholesterol [3,34], and StAR is a rate-limiting protein that mediates the transport of cholesterol from OMM to IMM. While HSD3B is an enzyme that converts pregnenolone to progesterone [2,3], its expression significantly decreased after DAPT treatment. The upregulation of NPC1 and StAR may override the downregulation of HSD3B protein on progesterone biosynthesis, resulting in upregulated progesterone secretion in pGCs.

We found that *NR5A2* and *NR2F2* reciprocally repressed Notch pathway signaling in pGCs. This result is similar to that of previous studies, namely, that NR5A2 and NR2F2 are involved in the Notch pathway, and the two TFs mutually inhibited the Notch pathway signaling in various systems [27,28]. Both the Notch effectors (Hes/Hey proteins) and NICD were reported to act as transcriptional repressors. For example, Hey inhibited the transcriptional activities of GATA4/GATA6 in mouse Leydig cells [11], cardiac cells [35,36], and mouse embryoid bodies [37]. NICD directly binds to CREB and inhibits its transcriptional activities in primary cortical neurons [38]. In pGCs, NICD and Hes/Hey proteins may inhibit NR5A2 and NR2F2 expression by suppressing the transcriptional activities of TFs for NR5A2 and NR2F2. On the other hand, NR5A2 was reported to inhibit Notch signaling by enhancing the expression of a TF, named Prox1, in neural stem cells [27]; NR2F2 suppresses NP-1 to downregulate Notch signaling in the endothelium of veins [39]. The mediators of NR5A2 and NR2F2 in regulating Notch signaling in pGCs are still unclear, and the detailed mechanism of how NR5A2 and NR2F2 reciprocally regulate Notch signaling still needs to be elucidated.

NR5A2, also known as liver receptor homolog-1 (LRH1), is highly expressed in the GCs of primary to preovulatory follicles and luteal cells [40], and it is essential for reproduction [22]. We found that *NR5A2* knockdown reversed the stimulatory effects of DAPT on progesterone secretion and the expression of NPC1 and StAR in this study. These results are supported by the phenotypes of *NR5A2* knockout mice [22,26,41,42]. Mice lacking NR5A2 are sterile, and the plasma progesterone concentration is seriously reduced due to a decreased StAR protein level [22,26,41,42]. The expression of *HSD3B* varied between different NR5A2 conditional knockout mice. In Cyp19a-Cre-mediated *NR5A2* conditional knockout mice, the expression of HSD3B was significantly upregulated [22], while in Amhr2-Cre- and Pgr-Cre-mediated *NR5A2* conditional knockout mice, the expression of *HSD3B* remained unchanged and reduced, respectively [26,42]. These results indicated that our in vitro *NR5A2* knockdown results were similar to the results in Cyp19a-Cre-mediated *NR5A2* conditional knockout mice.

NR2F2, also known as chicken ovalbumin upstream promoter transcription factor II (COUP-TFII), is an orphan nuclear receptor [28], and it plays important roles in development and steroidogenesis [28]. Our in vitro *NR2F2* knockdown results indicated that NR2F2 positively regulated the expression of NPC1 and StAR and progesterone secretion. These results are supported by the significant reduction in progesterone synthesis and gene expression of *StAR*, *Cyp11a1*, and *HSD3B* in NR2F2 haploinsufficiency female mice [43]. In MA-10 and MLTC-1 Leydig cells, NR2F2 activated steroidogenesis by stimulating *StAR* expression but not by regulating the expression of *HSD3B*, *Cyp11a1*, *Cyp17a1*, or *Cyp19a1* [44]. It is clear that NR2F2 acts as a TF that positively regulates steroidogenesis through the upregulation of the expression of steroidogenic proteins, particularly StAR.

In conclusion, we have described an inhibitory role of Notch signaling in progesterone biosynthesis. We further proved that Notch signaling reciprocally repressed the expression of NR5A2 and NR2F2 and the upregulation of NR5A2 and NR2F2 after the suppression of Notch pathway by DAPT treatment was responsible for the upregulation of progesterone secretion and the expression of NPC1 and StAR. Our study provided new insights into Notch signaling in steroidogenesis in ovary and raised new questions about the underlying mechanisms of the reciprocal repression between Notch signaling and the TFs NR5A2 and NR2F2.

## Supplementary Materials

The following are available online at https://www.mdpi.com/2073-4425/11/2/120/s1, Figure S1: Effects of DAPT treatment on the expression of Notch ligands and ERK1/2 phosphorylation, Figure S2: Gene expression of LDLR, VLDLR, and SRB1 after knockdown of NR5A2 and NR2F2 in pGCs, Table S1: Primers used for RT-qPCR, Table S2: Gene expression level of Notch proteins in porcine GCs.
