# Supplementary material for: Suppression of Notch Signaling Stimulates Progesterone Synthesis by Enhancing the Expression of NR5A2 and NR2F2 in Porcine Granulosa Cells
Source: Genes (Basel). 2020 Jan 22;11(2):120. doi: 10.3390/genes11020120 (PMC7073743; doi:10.3390/genes11020120)
Supplement: Supplementary file 1 [file genes-11-00120-s001.pdf]

genes

Supplementary Information

Suppression of Notch Signaling Stimulates Progesterone Synthesis by Enhancing the Expression of NR5A2 and NR2F2 in Porcine Granulosa Cells

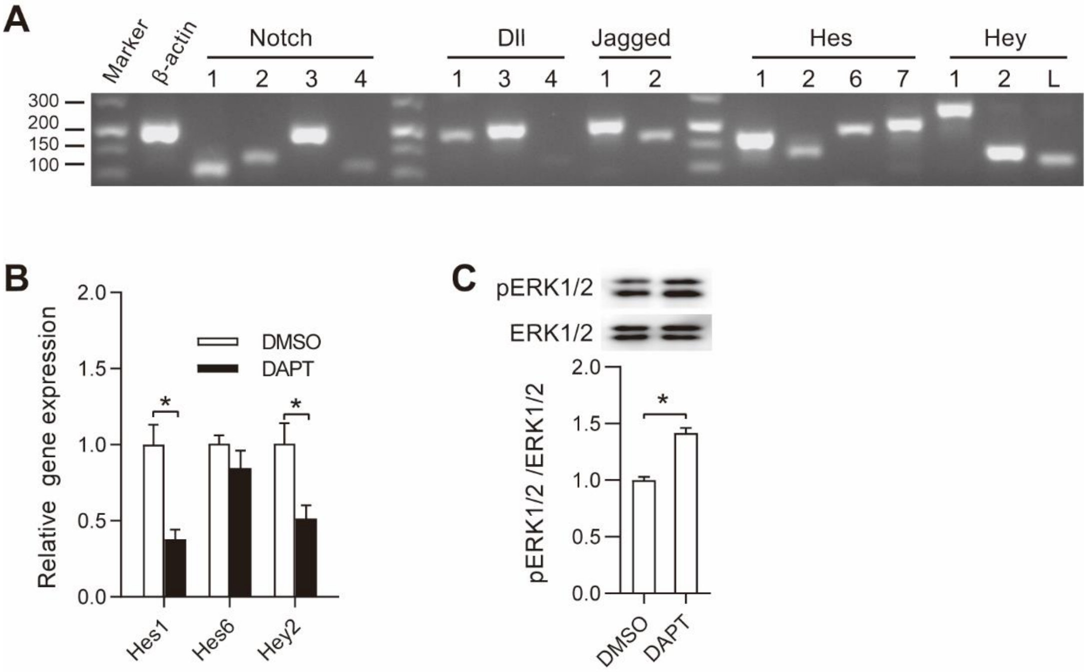

Figure 1. Effects of DAPT treatment on the expression of Notch ligands and ERK1/2 phosphorylation. (**A**) RT-qPCR products of four Notch receptors (Notch 1 –4), five canonical ligands (Dll 1, 3, 4 and Jagged 1, 2) and parts of Notch effectors (Hes and Hey proteins); (**B**) The expression of Notch ligands after DAPT treatment; (**C**) Proteins and phosphorylation of ERK1/2 in pGCs after DAPT treatment. pGCs were treated with 25 μM DAPT for 48 h. * means significant difference at $p < 0.05$.

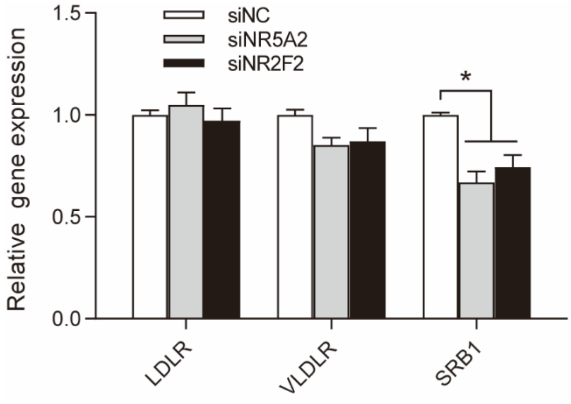

Figure 2. Gene expression of LDLR, VLDLR and SRB1 after knockdown of NR5A2 and NR2F2 in pGCs. * means significant difference at $p < 0.05$.

Table 1. Primers used for RT-qPCR.

Genes symbol	Primers (5'→3')'	GenBank accession no.	Length (bp)
Notch1	F: CCTACAAGATCGAGGCCGTG<br>R: ACGAAGAAGAGCAGCACGAA	XM_021081037.1	106
Notch2	F: TCTGCTCACCAGGATTCA<br>R: CCTCGGGGCACATACAAC	XM_021090689.1	126
Notch3	F: AGAGGCCAAACGACTGAAGG<br>R: CCATCTGGACCTCGCACATT	XM_021083631.1	186
Notch4	F: CTGTGAGGTGAACCCAGACG<br>R: ACACCCTGAGCCTCACATTC	NM_001123147.1	153
Jagged1	F：GTCTGTCTCTGCAGGTGTGG<br>R: TGCGCAGTTGTCCTGGTAAT	XM_021077352.1	209
Jagged1	F: GAGACGGTCGTCATAGGCAG<br>R: CTCCTCTCCCGCTCTTTCCT	XM_021081545.1	137
Dll1	F: TGAAGCTCTGCACACGGATT<br>R: AGTAGTGCTCGTCACACACG	NM_001244418.1	179
Dll3	F: GGTCTCATGCGTGTACCCTT<br>R: GGGCCCTCCAATCTGTTCTC	XM_013988565.2	196
Dll4	F: CTTCTTCCGCGTCTGCCTTA<br>R: CGTGCCAAGCTTCGATGATG	NM_001244418.1	203
Hes1	F: AACACGACACCGGATAAACCA<br>R: TTCTCCAGCTTGGAATGCCT	NM_001195231.1	167
Hes2	F: CACGTCCTCCGACAGCTAC<br>R: CAGGTGCTCCAACAGGCG	XM_003127529.4	118
Hes6	F: CAAAGAGAAGAACCGACTGCG<br>R: CGAAGGCTTTGCTGTGTTTCA	XM_003359692.4	188
Hes7	F: CTACTTGAGGGAGCGAAGCC<br>R: GGAAGCCGGACAAGTAGCAG	XM_021067858.1	98
Hey1	F: CATCATTGAGAAGCGCCGTC<br>R: TTCCCGAAACCCCAAACTCC	XM_005663011.3	235
Hey2	F: CGAAAACAATTACTCGGGGCA<br>R: CGATCTCGACGCCTTTTCTCTA	NM_001243329.1	123
HeyL	F: CGGACCGATTGACGTGGG<br>R: TCTGCGTTTCTTCCTGGCTT	XM_003127826	100
FoxL2	F: TTCGAGAAGGGCAACTACCG<br>R: TTGTTGAGGAAGCCGGACTG	NM_001244665.1	194
CREB1	F: AACATCATCTGCTCCCACCG<br>R: TCTGAGTTCCGGAGAAAAGTCT	NM_001361427.1	167
GATA4	F: GTCCCATCAAGACAGAGCCC<br>R: GACCGAAGATGCGTAGCCTT	NM_214293.1	158
GATA6	F: CCTCGACCGCTTGCTATGAA<br>R: GCTGGCGTTTGTGTTGTAGG	NM_214328.2	182
NR5A1	F: CAAGATCGACAAGACGCAGC<br>R: CCATTGGCTCGAATCTGTGC	NM_214179.1	186
NR5A2	F: GGTACCACTATGGGCTCCTCAC<br>R: TCGGCCCTTACCGCTTCT	NM_001267893.1	193
SREBF1	F: GCGACGGTGCCTCTGGTAGT<br>R: CGCAAGACGGCGGATTTA	XM_021091444.1	218
SREBF2	F: TTACCGAAGTGGAGCGTGTC<br>R: AAGGAACTCTGCTGCCCATC	XM_021091444.1	130
StAR	F: AACCTCAGGGTCATGGATT<br>R: CACTTTACTCAGCACCTCGTC	NM_213755.2	208
3βHSD	F: CCTTCAATCGCCACTTCG<br>R: CGCCTCCTTGTGCTGCTT	NM_001004049.2	161
Cyp11a1	F: CTGAACACGGAGGTAATGG<br>R: CAAAGGCGAAGCGAAACA	NM_214427.1	163
β-Actin	F: CTTCCTGGGCATGGAGTCC<br>R: GGCGCGATGATCTTGATCTTC	XM_003357928.4	201

Table 2. Gene expression level of Notch proteins in porcine GCs.

Notch protein	Symbol	Gene_ID	FPKM
Notch receptors	NOTCH1	LOC110258061	1.06
	NOTCH2	LOC100153369	38.67
	NOTCH3	LOC102158881	3.09
	NOTCH4	LOC100144522	0.08
Notch effectors	HES1	LOC100499567	15.26
	HES2	LOC100515666	0.03
	HES3	LOC100737699	0.05
	HES4	LOC100739264	0.36
	Hes5	LOC110261433	0.00
	HES6	LOC100622397	16.33
	HES7	LOC100620745	0.00
	HEY1	LOC100157952	0.14
	HEY2	LOC100152404	12.95
	HEYL	LOC100518256	0.01
Notch ligands	DLL1	LOC100620481	0.15
	DLL4	LOC100152163	0.00
	DLL3	LOC100520433	0.09
	JAG1	LOC100156773	3.52
	JAG2	LOC100522743	0.34
